# Anti-MDA5 dermatomyositis: an update from bench to bedside

**DOI:** 10.1097/BOR.0000000000000908

**Published:** 2022-09-12

**Authors:** Enrico Fuzzi, Mariele Gatto, Margherita Zen, Chiara Franco, Elisabetta Zanatta, Anna Ghirardello, Andrea Doria

**Affiliations:** Division of Rheumatology, Department of Medicine DIMED, Padua University Hospital, Padua, Italy

**Keywords:** anti-MDA5 antibodies, dermatomyositis, immunosuppressants, interstitial lung disease, rapidly progressive interstitial lung disease

## Abstract

**Purpose of review:**

This review summarizes the recent developments about anti-MDA5 antibody positive dermatomyositis with a focus on its pathogenesis, clinical features and treatment options of rapidly progressive interstitial lung disease, its most ominous complication.

**Recent findings:**

Anti-MDA5+ dermatomyositis has a heterogeneous clinical spectrum with different patient subsets exhibiting widely different outcomes; severe acute interstitial lung disease is the main factor impacting prognosis. The pathogenetic role of anti-MDA5 antibodies is an active area of investigation.

**Summary:**

Anti-MDA5+ dermatomyositis has a wider spectrum of manifestations than previously thought. A high index of suspicion is needed not to miss atypical presentations. In the setting of acute interstitial lung involvement, once a confident diagnosis is made, an aggressive approach with early combined immunosuppression affords the best chances of survival.

## INTRODUCTION

Immune-mediated inflammatory myopathies (IIM) are increasingly recognized as complex multisystem diseases with a wide spectrum of organ manifestations engendered in different proportions by inflammation, autoimmunity and vasculopathy [1,2,3]. The description and characterization of several myositis-specific and associated antibodies (MSAs and MAAs) has been a key contribution to defining different myositis clinical and pathophysiological subsets [4,5]. Among these, anti-melanoma differentiation antigen 5 (MDA5) antibodies have been associated with a definite subset of dermatomyositis patients showing prominent cutaneous and lung involvements with rapidly progressive interstitial lung disease (RP-ILD). The spectrum of anti-MDA5+DM is being explored further and subdivided into different clinical and prognostic subsets. Anti-MDA5 antibodies may also be found in the context of isolated lung involvement [6]; thus, the term ‘anti-MDA5 syndrome’ has been recently proposed [7^▪▪^].

Furthermore, a hyperinflammatory and hyperferritinemic state can be documented at the time of clinical worsening in some of these patients, bearing resemblance to severe cases of human SARS-CoV2 infection [8–11].

In contrast with classical forms of dermatomyositis, no strong association is consistently reported between MDA5+DM and malignancy. Recent research acquisitions have focused on describing the clinical spectrum associated with anti-MDA5 antibodies in Asian and non-Asian settings, in identifying predictors of RP-ILD and death, and on a deeper understanding of anti-MDA5 antibodies, whether as a directly pathogenic entity or as a marker of an underlying pathological process. 

**Box 1:**
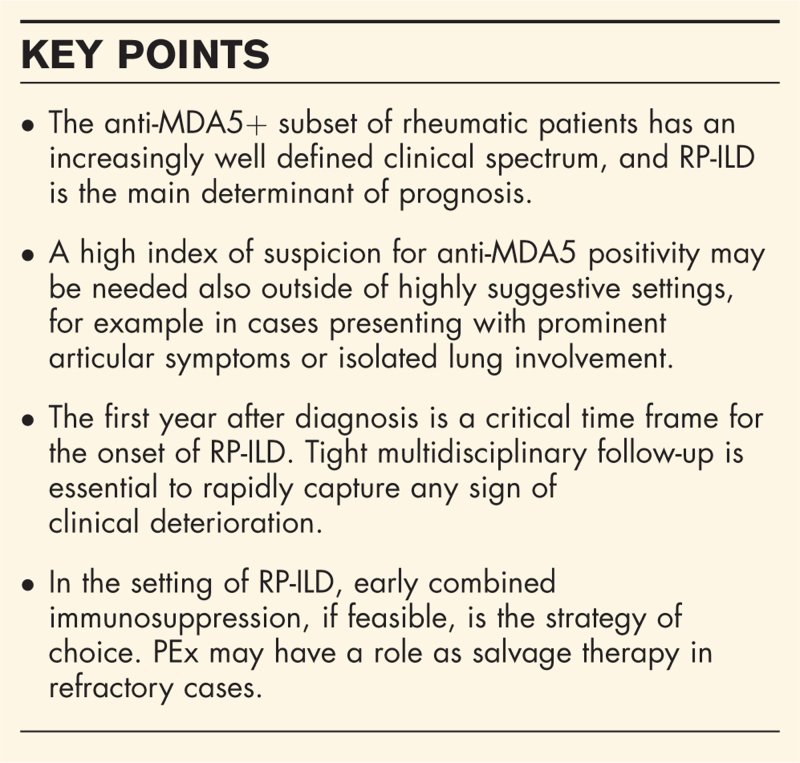
no caption available

## THE BENCH: MDA5 AND ANTI-MDA5 ANTIBODIES

Originally described in melanoma cells and thence deriving its namesake, MDA5 is an antiviral pattern recognition receptor in humans. MDA5 is a cytosolic receptor that recognizes long strands of double-stranded RNA, a foreign molecular structure in eukaryotic cells. The origin of such molecules stems mainly from RNA viruses and DNA viruses, but dsRNA can also have an endogenous mitochondrial origin. Upon binding to dsRNA, through interaction with mitochondrial antiviral signalling protein (MAVS), MDA5 enhances the transcription of interferon-dependent (IFN) genes. In turn, MDA5 itself is encoded by a IFN-inducible gene (IFIH-1). Therefore, MDA5 sits at the origin of a positive proinflammatory and interferogenic feedback loop, occupying a critical regulatory position.

Hyperfunction of MDA5 due to gain-of-function mutations results in a spectrum of diseases sharing malformations, chronic inflammation and features of an interferonopathy with several rheumatological manifestations [12,13]. Furthermore, hyperstimulation of MDA5 by defective clearance mechanisms for mitochondrial dsRNA – for example in hypomorphic polynucleotide phosphorylase mutations – also results in an interferonopathy [14]. Importantly, the range of MDA5 subcellular localizations is not yet entirely clear: indeed, although MDA5 is classically described as a cytosolic receptor, it may relocate when abundant [15]. An overexpression of MDA5 in response to an index event may promote a shift in its subcellular localization, and it may encourage loss of tolerance to MDA5 and production of anti-MDA5 antibodies.

**FIGURE 1:**
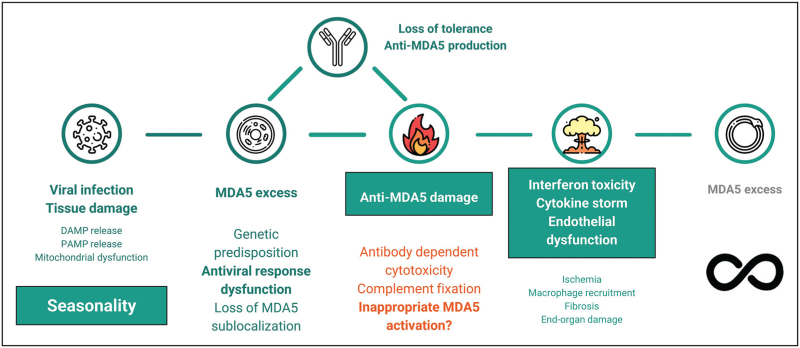
Proposed general pathogenetic model of the anti-MDA5 syndrome. DAMP, damage-associated molecular pattern; MDA5, melanoma differentiation antigen 5; PAMP, pathogen-associated molecular pattern. Icons made by Freepik from Flaticon.com.

Anti-MDA5 could exert pathological effects on both ends of their functional spectrum: Anti-MDA5 antibodies that inactivate MDA5 may compromise antiviral responses, altering them to the point of indirectly producing an excessive, inefficient and damaging multisystemic inflammation to sustain viral clearance. On the opposite end, anti-MDA5 antibodies may stabilize MDA5 in an ‘active’ configuration, thus creating a constant danger signal at the origin of a pernicious positive feedback, producing the same hyperinflammatory state [16]. Several other mechanisms may be implicated in a direct anti-MDA5-mediated damage, such as formation of immune complexes together with MDA5, cell penetration with downstream pathway disruptions and antibody-dependent cytotoxicity. Anti-MDA5 could also simply be a marker of a dysfunctional antiviral response, with overexpression of MDA5 and loss of tolerance towards it as an epiphenomenon. However, it is increasingly clear that not all anti-MDA5 antibodies are made equal: in a recent study, Anti-MDA5 IgG-1 were found to be associated with RP-ILD and Anti-MDA5 IgA were found to be common, while the IgM isotype was more unusual [17]. In a different study, IgG1 and IgG3 anti-MDA5 antibodies were found to be independently associated with death and with RP-ILD, in contrast with IgG2 and IgG4 [18]. Titres of anti-MDA5 antibodies also seem to be higher in nonsurvivors and in RP-ILD patients, although this is not a universal finding [19,20]. Therefore, anti-MDA5 antibodies have potential roles both as *markers* and *makers* of a potentially devastating disease. In a general pathogenetic model (Fig. 1): an index event – presumably a viral infection – is met by a genetically susceptible host with an exuberant production of MDA5, loss of its subcellular localization, tissue damage and break of tolerance. A late immune response with delayed IFN production may promote this maladaptive process, whereas a rapid and orderly virus clearance through a timely initial burst of IFN production may avert further complications, in a similar manner to that described in COVID-19 [21,22]. Anti-MDA5 antibodies, once produced, may further exacerbate the process, leading to more inflammation and tissue damage, and engendering a cytokine storm in which high levels of IFN may mediate a vasculopathy through endothelial toxicity [23,24]. The healing response to the ongoing damage and ischemia would promote macrophage recruitment [25], fibrosis [26] and irreversible organ damage, especially in the lungs.

This conceptual framework bears several similarities with the human infection by SARS-CoV-2. Of note, anti-MDA5 antibodies have been found in COVID-19 patients, and their presence and titre showed an association to mortality [27]. On the contrary, nonspecific positive antibody tests are commonplace during viral infections, and anti-MDA5 titres were rather low compared with true anti-MDA5+DM patients.

## THE BEDSIDE: CLINICAL CLUSTERS

The first descriptions of anti-MDA5+ dermatomyositis entailed a combination of clinically amyopathic dermatomyositis (CADM) with RP-ILD [28,29]. The cutaneous manifestations included hallmarks of dermatomyositis such as heliotrope rash, Gottron's papules and sign, and other typical dermatomyositis rashes such as V-neck and shawl signs. The presence of prominent cutaneous vasculopathy with skin ulcers was also an outstanding clinical feature.

Since then, the picture has evolved with the availability of retrospective data from both Asian and non-Asian cohorts [30–33]. In a recent unsupervised analysis on a French nationwide multicentre retrospective cohort [34], three clinical phenotypes were proposed: a ‘rheumatoid cluster’ exhibiting mostly arthritis and dermatologic involvement, with infrequent RP-ILD, a female predominance and a good overall prognosis; a male-predominant ‘vasculopathic DM cluster’ displaying severe vasculopathy in the form of Raynaud's phenomenon, skin ulcers and necrosis in addition to typical dermatomyositis rashes; in this group, rates of RP-ILD were intermediate (22.7%), as was the overall prognosis. Clinically relevant myositis (proximal weakness and high creatine kinase) was more prevalent in this subgroup. A ‘RP-ILD cluster’ with a grievous prognosis, high prevalence of ICU admission and very high rates of RP-ILD and death.

Some of these clusters are similar to other reports. In a recent single-centre retrospective Chinese cohort [35], three clusters emerged of which two were comparable to the French study: one mainly showing arthritis and mechanic's hands with low rates of RP-ILD and a good prognosis; one enriched in RP-ILD which was also exhibiting fever, hyperferritinemia and a far worse prognosis. In contrast, a different third cluster identified patients with high rates of typical cutaneous signs and enriched in clinically relevant myositis, with very low rates of RP-ILD (Table 1) [65].

**Table 1:** Focus on recent descriptive cohorts and salient clinical characteristics of anti-MDA5+DM and non-DM patients

Reference	Salient clinical involvement	RP-ILD rate	Prognosis	Comment
Allenbach *et al.n* = 121[39]	Cluster 1ILD 100%Skin 100%• mechanic's hands 73.3%	RP-ILD 93.3%	3-month mortality 80%	
	Cluster 2Skin 82.6%• Skin ulcers 37%ILD 82.6%Arthritis/arthralgia 82.6%	RP-ILD 17.4%	3-month mortality 0%	
	Cluster 3Skin 95.4%• Skin ulcers 77.3%• Digital necrosis 31.8%Raynaud phenomenon 81.8%Proximal weakness 68.2%ILD 50%	RP-ILD 22.7%	3-month mortality 4.5%	
Yang *et al.n* = 96[35]	Cluster 1Arthritis 84.6%Mechanic's hands 51.3%	RP-ILD 7.7%	24-week mortality 2.6%	
	Cluster 2V-neck sign 69.2%Muscle weakness 92.3%	RP-ILD 7.7%	24-week mortality = 0	
	Cluster 3Fever 77.3%Elevated CRP 100%Hyperferritinemia > 1000 ug/L 75%	RP-ILD 77.3%	24-week mortality 54%	
Cavagna *et al.n* = 149[7^▪▪^]	OverallSkin involvement 74%Symptomatic muscle involvement 49%Joint involvement 51%• symmetric polyarticular in 70%Skin ulcers 15%Raynaud phenomenon 30%Fever 29%	RP-ILD 21.5%	17% mortality at 36 months• 42% directly due to RP-ILD• 19% due to infection superimposed on RP-ILD	Focused on Anti-MDA5+ overall (10% diagnosed with IPAF)
	At presentationSkin alone 14%Skin + ILD 13%			
Hensgens *et al.n* = 20[65]	OverallILD 95%Skin findings 87%Arthritis/arthralgia 60%	RP-ILD 45%	1-year mortality 45%	Higher Anti-MDA5 titres in RP-ILD although with shorter disease duration

The rates of RP-ILD, overall or in different clusters depending on the study, are reported. CRP, C-reactive protein; FU, follow-up; ILD, interstitial lung disease; IPAF, interstitial pneumonia with autoimmune features; RP-ILD, rapidly progressive interstitial lung disease.

In a retrospective analysis of the AENEAS group focusing on anti-MDA5+ patients as a whole [7^▪▪^], 89% of patients were diagnosed with myositis (dermatomyositis 43%, CADM 31%, polymiositis 5%, overlap myositis 11%); interestingly, the remainder 10% was diagnosed with interstitial pneumonia with autoimmune features (IPAF), not satisfying any other classification criterion. ILD was the main manifestation (72%); skin, joint and muscle involvement also showed a significant prevalence (74, 51 and 56%, respectively). Notably, rates of RP-ILD (21.5%) were lower than in Japanese reports, but in line with other European reports [32]. Onset of ILD was not confined to the first stages of the disease, but it could be diagnosed after a long course and, importantly, after prior treatment with potent immunosuppressants. Although the methodology differs, clinical clusters were not as clear-cut in this study, and arthralgia/arthritis and Raynaud phenomenon did not show a clear segregation in particular subgroups. Importantly, more than half of the patients did not show a positive antinuclear titre, stressing the need to actively look for Anti-MDA5 antibodies whenever clinical suspicion arises.

In severe cases, the disease may be complicated by signs of an hyperinflammatory, hyperferritinemic syndrome similar to severe COVID-19 [8]; this subset is often represented by acutely ill patients with RP-ILD, peripheral cytopaenias, high ferritin, elevated liver enzymes and haemostatic imbalances with both bleeding events and a proclivity towards disseminated intravascular coagulation. For example, spontaneous intramuscular haemorrhages have been described in acutely ill anti-MDA5+ patients, often carrying a grave prognosis [36]. Some of these severe cases may satisfy criteria for macrophage activation syndrome [37], including the presence of haemophagocytosis at bone marrow examination [38]. Awareness of such haematologic manifestations as part of the clinical picture is of critical importance, because these may otherwise lead clinicians astray in what appears to be a time-sensitive and difficult-to-treat disease.

Taken together, recent evidence suggests that any patient presenting with a suspicion or a known positivity for anti-MDA5 antibodies should prompt the treating physician to perform an assessment of a full patient history and a thorough examination of skin, muscle, joints and lungs; chest imaging with high-resolution computed tomography (HRCT) should be obtained expeditiously if any clinical signs of lung involvement are present; if not, at least pulmonary function tests (PFTs) and first-line chest imaging are advisable. Once any level of lung involvement is diagnosed, appropriate therapy and a tight multidisciplinary follow-up by Rheumatology, Pneumology and, if possible, Radiology should be arranged.

## INTERSTITIAL LUNG DISEASE, RAPIDLY PROGRESSIVE- INTERSTITIAL LUNG DISEASE AND PREDICTORS OF POOR OUTCOME

RP-ILD is the main factor impacting prognosis in anti-MDA5+DM. Although ILD and RP-ILD can ensue at any point in the disease course, RP-ILD peaks in the first 6–12 months from diagnosis, and it drives mortality in this early period [39,40]. Predictors of both RP-ILD and mortality are therefore of great clinical interest.

The available data, derived from multivariate analyses of retrospective cohorts, point to the following factors as independently associated with ILD in the setting of anti-MDA5+DM: older age, a high neutrophil-to-lymphocyte ratio and/or lymphopenia, elevated LDH, elevated ferritin. The exact ferritin cut-off is variable among studies, with the majority reporting levels in excess of 1000 μg/l. Fever and elevated CRP have also been implicated in portending a worse prognosis (Table 2) [66–68]. These thought-provoking findings reinforce the notion of a dysfunctional antiviral response or a cytokine storm as the underlying substrate of the disease, at least in severe cases.

**Table 2:** Focus on recent studies reporting on associated factors to rapidly progressive interstitial lung disease and mortality in Anti-MDA5+DM

References	Outcome	Risk factors (except RP-ILD)
Zuo *et al.*[43]	RP-ILD	Fever OR 3.672 (1.794–7.516)Elevated ALT OR 2.355 (1.153–4.813)Elevated LDH OR 3.083 (1.517–6.266)Lymphopenia OR 2.141 (1.013–4.528)Elevated Ferritin OR 4.965 (1.973–12.498)Elevated CEA OR 2.276 (1.128–4.591)Elevated CA 15.3 OR 3.305 (1.502–7.272)Protective:Arthralgia OR 0.281 (0.138–0.570)
	Mortality	Ferritin > 2200 ng/ml AUC 0.66 (0.51–0.80)
So *et al.*[66]	RP-ILD	Age > 50 years HR 2.640 (1.277–5.455)LDH > 300 U/L HR 3.189 (1.469–6.918)Fever HR 1.903 (0.956–3.790)NLR > 7 HR 1.967 (0.942–4.107)
	Mortality	Age > 52 years HR 4.750 (1.692–13.333)LDH > 400 U/L HR 2.290 (1.009–5.198)Ferritin > 2800 pmol/l HR 3.042 (1.323–6.997)
Ouyang *et al.*[44^▪^]	Mortality	Fever HR 24.6 (2.3–260.7)Ferritin > 1250 μg/l HR 51.1 (3.5–747.5)Elevated CEA HR 85 (1.1–6516.2)
Zhou *et al.*[67]	Mortality	Advanced ageLymphopeniaLow serum albuminHigh LDHHigh ferritin
Lian *et al.*[68]	Mortality^a^	Ferritin > 636 ng/ml HR 2.62 (1.18–5.83)LDH > 355 U/l HR 3.59 (1.83–7.01)HRCT score HR 6.24 (1.47–12.56)

Where available, adjusted ORs, hazard ratios, AUCs and 95% confidence intervals are reported. AUC, area under the curve; CA15.3, Cancer-Antigen 15.3; CEA, carcinoembryonic antigen; HR, hazard ratio; HRCT, high-resolution CT; LDH, lactic dehydrogenase; NLR, neutrophil-to-lymphocyte ratio; OR, odds ratio.

a Analysis on a cohort of CADM-ILD patients, with Anti-MDA5+ as a subset.

The co-presence of anti-Ro52 (SSA) antibodies has repeatedly been reported to be enriched in ILD and RP-ILD patients [41,42], confirming the not-so-benign profile of this antibody in the setting of autoimmune lung involvement. In recent reports, higher peripheral CD5-CD19+ B-cell counts and elevated carcinoembryonic antigen (CEA) and CA 15.3. were remarked on as independently associated with RP-ILD [43], in addition to the previously mentioned factors. Moreover, in a recent matrix prediction analysis [44^▪^], three factors (ferritin, CEA, fever) successfully predicted mortality at 6 months. The elevation of oncomarkers may raise suspicion of malignancy being implicated: conversely, CEA levels are heightened in many forms of lung injury such as in idiopathic pulmonary fibrosis and in active smokers [45]; moreover, no cases of adenocarcinoma were reported by the authors at extended follow-up in patients with elevated CEA who survived. Radiological patterns vary between reports but frequently show a combination of nonspecific interstitial pneumonia (NSIP) and organizing pneumonia findings with basal involvement and a rapidly progressive consolidative pattern [46,47]; a UIP-like pattern has also been reported [7^▪▪^]. Quantification of lung involvement at HRCT contributes to inform prognosis [48–50].

Importantly, although radiology may offer some crucial clues during the diagnostic stage, it remains challenging for any single radiological pattern to uniformly clinch the diagnosis of anti-MDA5 lung involvement *a priori* without supporting clinical and serological evidence; this reinforces the importance of actively looking for anti-MDA5 antibodies whenever clinically indicated.

## THERAPEUTIC DEVELOPMENTS in RAPIDLY PROGRESSIVE-INTERSTITIAL LUNG DISEASE

No universal recommendations exist for treatment of anti-MDA5+DM. Outside of RP-ILD, current therapies are targeted towards the prevailing clinical manifestations whether it be arthritis, myositis, cutaneous rashes and vascular/vasomotor manifestations. In observational studies, employed drugs include glucocorticoids, antimalarials, methotrexate, mycophenolate mofetil, calcineurin inhibitors and azathioprine [7^▪▪^]. Intravenous immunoglobulins (IvIGs) and rituximab also have a role, especially as second-line interventions.

In the setting of RP-ILD, glucocorticoids in isolation do not seem to offer benefit and recent evidence supports early combined immunosuppression, with a low threshold for therapy escalation, and consideration to therapeutic plasma exchange (PEx) as salvage therapy in unresponsive cases (Table 3) [51,52^▪^]. The main strategy, supported by retrospective and prospective data, entails the combined use of high-dose glucocorticoids, for example intravenous methylprednisolone pulses 500 mg to 1 g/day for at least three consecutive days followed by 1 mg/kg/day, a calcineurin-inhibitor (CNI) and intravenous cyclophosphamide (CYC) 0.5–1.0 g/m^2^. In Japanese studies, early combination therapy yielded a better survival rate when compared with step-up therapy [53,54^▪^]. PEx could afford some incremental survival in cases not responding to combination therapy [55^▪^,^56^]. Of note, PEx outside of a combined immunosuppressive regimen appears to be of little value [54^▪^]. Combination therapy with glucocorticoids and a CNI, especially Tacrolimus, without CYC may yield similar results to triple therapy [57]. Among CNIs, Tacrolimus may perform better than Cyclosporin A [58]. Retrospective evidence suggests that the use of Rituximab as an add-on therapy to background immunosuppression could be a valid option [59]; an ultra-low dose regimen (100 mg single dose) also showed a nonstatistically significant trend towards response [60].

**Table 3:** Focus on selected key recent evidence on treatments of Anti-MDA5+-ILD. Studies employing control groups are reported

References	Design and intervention	Study population	Result
Shirakashi *et al.*[55^▪^]	Retrospective case-controladd-on PEx vs. no PEx	Anti-MDA5+ RP-ILD*n* = 38of which progressing under combined immunosuppression*n* = 13	3-year survival of 62.5% in PEx group vs. 0% in no PEx group (*P* = 0.04, significant)
Abe *et al.*[56]	Retrospective case-controladd-on PEx vs. no PEx	Anti-MDA5+ RP-ILD under combined immunosuppression*n* = 10	1-year survival 100% in PEx group vs. 25% in no PEx group (*P* = 0.033, significant)
Mao *et al.*[60]	Retrospective case-controlsingle 100 mg RTX infusion with or without CYC vs. CYC	Anti-MDA5+ ILD, RP-ILD in 92.5%*n* = 40	180-day mortality 36.4% in RTX group vs. 63.2% in CYC alone group (*P* = 0.26, nonsignificant)
Tsuji *et al.*[54^▪^]	Prospective single-arm with historical control groupCombined immunosuppression vs. traditional high-dose GCs with or without add-on PEx	Anti-MDA5+ ILD*n* = 44	12-month survival 85% in combined immunosuppression group vs. 33% in traditional immunosuppression(*P* < 0.001, significant)12-month survival 85% in add-on PEx vs. 71% in no add-on PEx (*P* = 0.17, nonsignificant)
Fujisawa *et al.*[58]	Prospective, randomized open-label 52 weeks trialTacrolimus vs. Cyclosporine	Myositis-associated ILD, subgroup for Anti-MDA5+ patients*n* = 58	Survival 88% in TAC group vs. 80% in CsA group (*P* = 0.63, nonsignificant)Progression-free survival 63% in TAC group vs. 40% in CsA group (*P* = 0.32, nonsignificant)
Chen *et al.*[63]	Prospective open-label with historical control groupTofacitinib vs. no Tofactinib	Anti-MDA5+ ILD, early (< 3 months)*n* = 50	6-month survival of 100% in Tofacitinib group vs. 78% in control group (significant at *P* = 0.04)

CYC, cyclophosphamide; PEx, plasma exchange; RTX, rituximab.

Apart from PEx, other salvage therapies include Polymyxin B Hemoperfusion, which unfortunately has not shown encouraging results [61]. Extracorporeal membrane oxygenation (ECMO), while not a disease-modifying therapy *per se*, can act as a bridge to recovery or bridge to transplantation through the most critical stages of lung dysfunction [62].

Obviously, an aggressive combined immunosuppression has the drawback of being at odds with the main other confounding factor at the diagnostic and follow-up stages: infection. In fact, infections remain an important cause of death in anti-MDA5+DM patients [7^▪▪^]. A swift microbiologic workup and close collaboration and shared decision-making between different specialist figures are therefore key to avert unfavourable outcomes in this difficult disease.

Lastly, JAK inhibitors have been reported to be effective, especially in early cases [63]. Isolated reports of a combined use of JAKis with RTX with good effect are also available [64]. Further controlled studies are needed to properly assess the treatment hierarchy.

## CONCLUSION

The spectrum of disease manifestations associated with anti-MDA5 antibodies is complex and expanding. Anti-MDA5+DM encompasses different patient groups with different prognoses, with RP-ILD being the main prognostic watershed. Several challenges lie ahead, including obtaining a better understanding of the role of anti-MDA5 antibodies, and achieving clarity on which treatment is the most indicated within and outside the setting of RP-ILD. Collaboration between the different medical specialties of Rheumatology, Pulmonology, Intensive Care and Radiology is paramount to achieve better outcomes.

## Acknowledgements

*The authors would like to thank Francesca Faustini, Antonella Notarnicola and Lara Dani (Rheumatology Unit, Karolinska Institutet, Stockholm, Sweden) for useful discussions and comments.*

### Financial support and sponsorship

*None.*

### Conflicts of interest

*There are no conflicts of interest.*

## REFERENCES AND RECOMMENDED READING

Papers of particular interest, published within the annual period of review, have been highlighted as:

▪ of special interest
▪▪ of outstanding interest
